# Supplementary material for: Pharmacy Students’ Perceptions and Stigma Surrounding Naloxone Use in Patients with Opioid Use Disorder: A Mixed Methods Evaluation
Source: Pharmacy (Basel). 2020 Nov 3;8(4):205. doi: 10.3390/pharmacy8040205 (PMC7712231; doi:10.3390/pharmacy8040205)
Supplement: Supplementary file 1 [file pharmacy-08-00205-s001.pdf]

An exploratory study of pharmacy students' perceptions of opioid prescribing and naloxone knowledge

Principal Investigator:

Alina Cernasev, PharmD, PhD

Co-Investigators:

Kenneth C. Hohmeier, Pharm.D., Director of Community Affairs

Michael Veve, Pharm.D., MPH

Elizabeth Hall, Pharm.D.

Q1 How would you describe your overall health?

(1) Excellent
(2) Very Good
(3) Good
(4) Average
(5) Poor

Q2 How satisfied are you with your dental health?

(1) Extremely satisfied
(2) Moderately satisfied
(3) Slightly satisfied
(4) Neither satisfied nor unsatisfied
(5) Slightly unsatisfied
(6) Moderately unsatisfied
(7) Extremely unsatisfied

Q3 How would you rate your physical health?

(1) Excellent
(2) Very Good
(3) Good
(4) Average
(5) Poor

Part 1: Opioids

4) Are you currently a pharmacy intern?
   (1) Yes
   (2) No

5) If yes, in what setting(s) are you working?
   (1) Retail
   (2) Hospital
   (3) Long term facility
   (4) Mail order
   (5) Other

6) How long have you been working at your current pharmacy-related job?

7) When working in a pharmacy as an intern, how often are you asked by your patients if the pharmacy carries certain opioids or has certain quantities of opioids in stock?
   (1) Weekly
   (2) Monthly
   (3) Quarterly
   (4) Never

8) How often do you interact with patients receiving opioid prescriptions ?

   (1) Weekly
   (2) Monthly
   (3) Quarterly
   (4) Never

17) For each of the following statements, please indicate the extent to which you agree or disagree. Answers: (1) Strongly agree, (2) Disagree, (3) Somewhat agree, (4) Agree, (5) Strongly disagree

Q1: When there is not a clear solution for a patient with addiction, the pharmacy team usually consults with a physician.

Q2: When I suspect the dose is too high for an opioid prescription, I usually consult with the pharmacist to further address it.

Q3: Information about a patient's prescription history is important to know when filling opioid prescriptions.

Q4: When counseling on opioid prescriptions, I usually use a loud voice, and words such as "abuse" or "overdose" to explain the risk of addiction to the patient.

Q5: When I work as an intern, I believe that patients coming to fill multiple opioid prescriptions might have developed addiction.

Q6: When a patient picks up an opioid prescription, it has no impact on my interaction with him/her.

Q7: When a patient picks up a prescription for naloxone, it does not influence the way I interact with him/her.

Q8: When I work as an intern, I never think of the fact that patients have been diagnosed with addiction.

Q9: Most of my colleagues believe that patients with multiple opioid prescriptions might show signs of addiction disorder.

Q10: It is important for me to participate in additional courses focused on opioid addiction and treatment care options to better serve my future patients.

<u>Part 3: Stigma Manifestation</u>

18) What is your comfort level in continuing a friendship if you found out your colleague in your class is abusing opioids?
(1) Very comfortable
(2) Somewhat comfortable
(3) Neutral
(4) Somewhat Uncomfortable
(5) Very uncomfortable

19) How likely would you be to avoid your colleague if you found out he/she were abusing opioids?
(1) Very likely

(2) Likely
(3) Neutral
(4) Unlikely
(5) Very unlikely

20) How likely would you shake the hands and explain the importance of "addiction-free" to a patient who usually picks up multiple opioid prescriptions?
(1) Most likely
(2) Likely
(3) Neutral
(4) Unlikely
(5) Very unlikely

21) How often is there mutual respect between pharmacy staff and patients who have misuse opioid prescriptions?
(1) Daily
(2) Several times a week
(3) 2-3 times a week
(4) Once a week
(5) Never

Part 4: Demographics

22) What is your age in years?

23) Which of the following apply for you? P1-P4

24) What is your sex? Female/ Male

25) How many years have you lived in the community you currently work in?

26) Did you work in a retail setting prior to pharmacy school? Yes/No

27) If yes, in which state did you work?

28) Before starting pharmacy school, what was the zip code for your county?

29) Which of the followings best describes your ethnic background?
(1) Asian/Pacific Islander
(2) Black/African American
(3) Native American

(4) White
(5) Other: Please Specify
(6) Prefer not to disclose
